# Supplementary material for: Simulating PIP2-Induced Gating Transitions in Kir6.2 Channels
Source: Front Mol Biosci. 2021 Aug 10;8:711975. doi: 10.3389/fmolb.2021.711975 (PMC8384051; doi:10.3389/fmolb.2021.711975)
Supplement: Supplementary file 2 [file DataSheet1.PDF]

Supplementary Material

1 Supplementary Figures

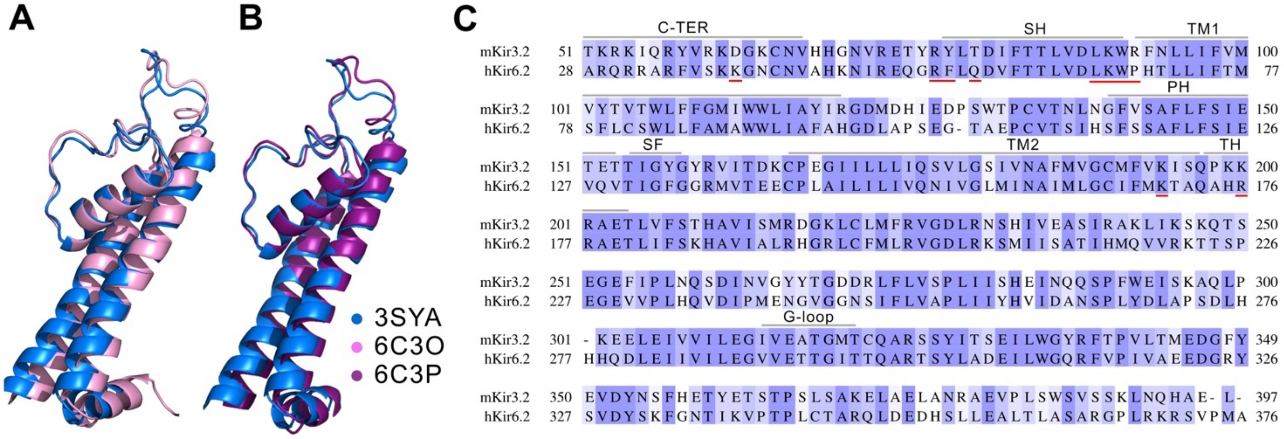

Supplementary Figure 1. Structure and sequence similarity between PIP_2 binding sites in Kir3.2 (3SYA) and Kir6.2 (6C3O, 6C3P). Structural alignment between (A) 3SYA and 6C3O (RMSD: 0.7 Å) and (B) 3SYA and 6C3P (RMSD: 0.5 Å) TMD regions. Proteins were aligned along the backbone with the Swiss-PdbViewer (Guex & Peitsch, 1997) and rendered with Pymol (Schrödinger, 2015). (C) The sequence alignment of mouse Kir3.2 and human Kir6.2 was colored according to the level of conservation in shades of blue. Kir6.2 residues forming the PIP_2 binding site, as determined by PyLipid (Suppl. Figure 8), are underlined in red.

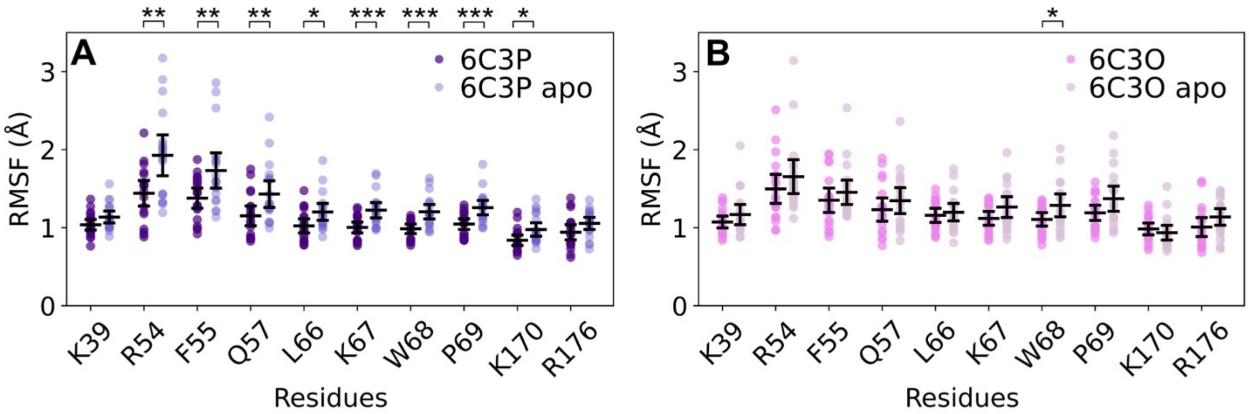

Supplementary Figure 2. Dynamics of residues in the PIP_2 binding site. Root-mean-square fluctuations (RMSF) are shown for systems (A) 6C3P and (B) 6C3O, each in the presence and absence (apo) of bound PIP_2. Relevant residues were chosen based on the PyLipid analysis (Suppl. Figure 8). For each residue, the first 200 ns of five trajectories comprising four protein subunits were analyzed with Gromacs (Abraham et al., 2015, 2018), resulting in 20 data points per residue. Error bars indicate the 95% confidence interval around the mean. Plots were generated with Matplotlib (Hunter, 2007), and the two-sided T-test was performed with SciPy (Virtanen et al., 2020): * $p < 0.05$, ** $p < 0.01$, *** $p < 0.001$

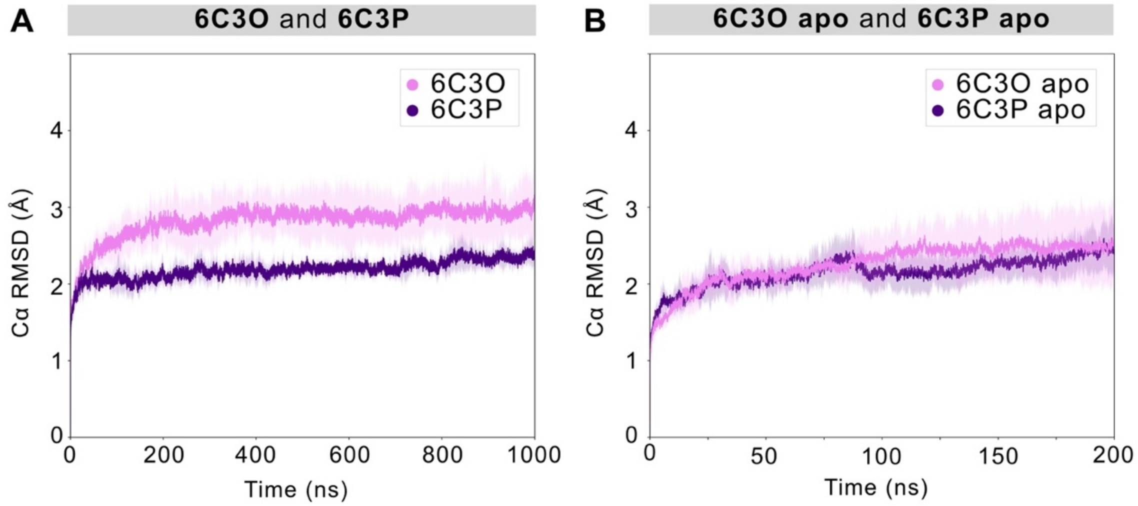

Supplementary Figure 3. Overall protein stability. Average root-mean-square deviations (RMSD) +/- SD of protein Cα atoms are shown for (A) Kir6.2 systems including PIP_2, and (B) control simulations without bound PIP_2 (apo) after excluding the highly flexible extracellular loop (r97-110) and intracellular C-terminus (r346-359) in Kir6.2. Averages were calculated of five replicas per simulation system.

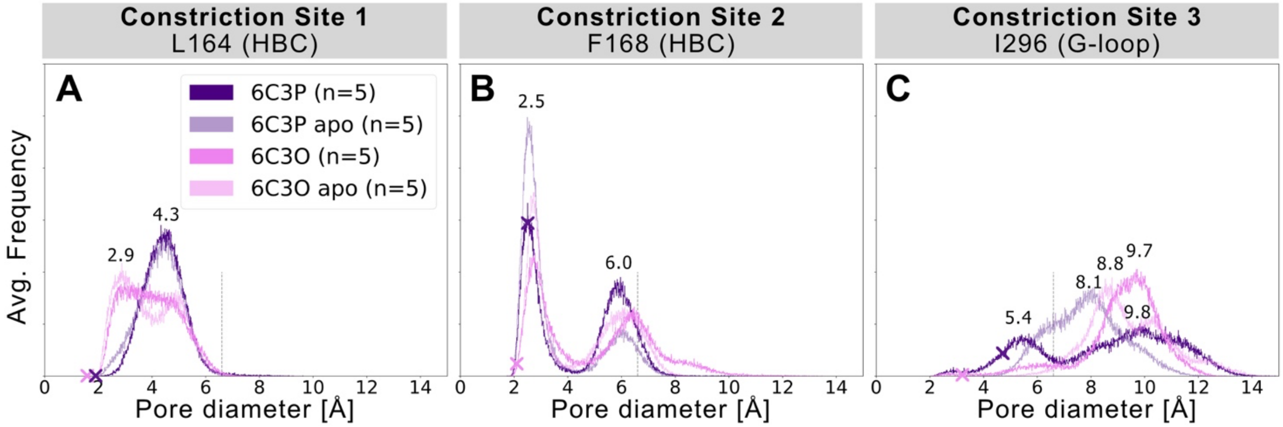

Supplementary Figure 4. Comparison of gate minimum distances in PIP_2-holo and apo systems. Minimum distances for three major constriction sites in Kir6.2 were measured in 5 x 200 ns MD simulation for all systems between two opposing subunits and subsequently averaged over the number of simulations. As shown in Figure 2, crosses mark the corresponding distance in the initial state of the cryo-EM structures before equilibration and production run, measured with the HOLE program. A vertical line is drawn at 6.6 Å, indicating the time-averaged hydration diameter of K+ (Conway, 1981).

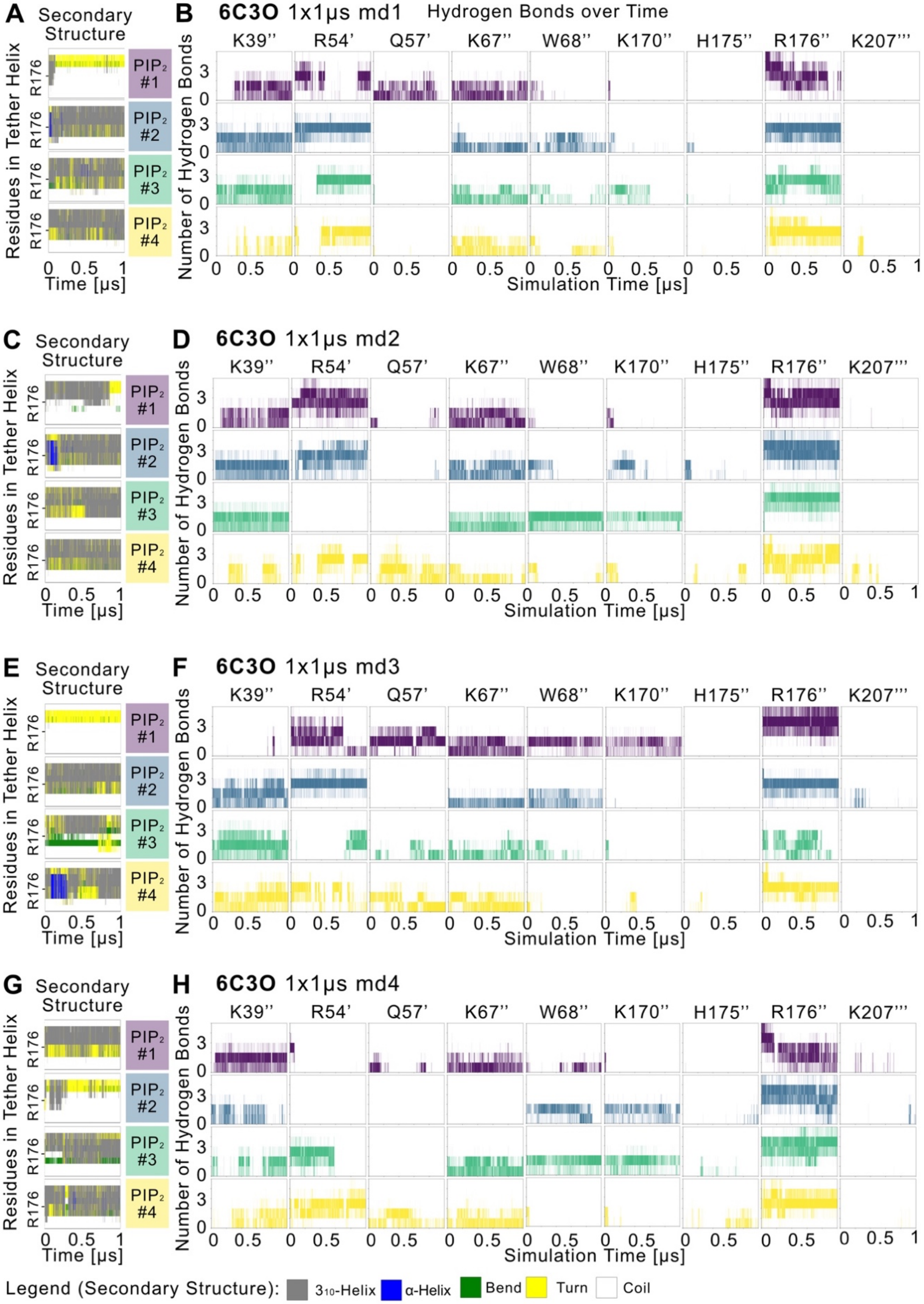

Supplementary Figure 5. PIP_2 binding site and PIP_2-induced gating changes for the remaining 6C3O simulations. For a description, please see Figure 3.

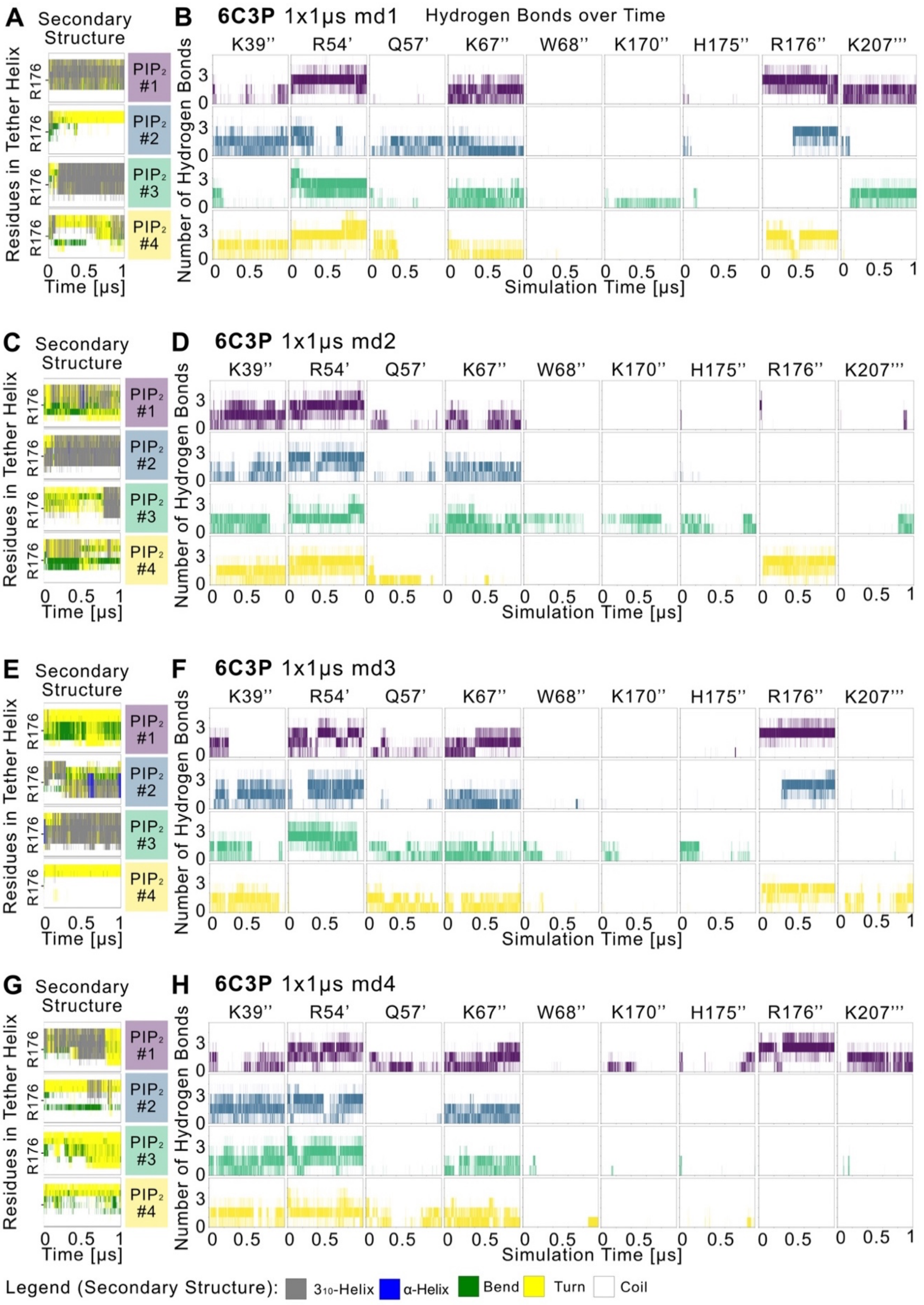

Supplementary Figure 6. PIP$_2$ binding site and PIP$_2$-induced gating changes for the remaining 6C3P simulations. For a description, please see Figure 3.

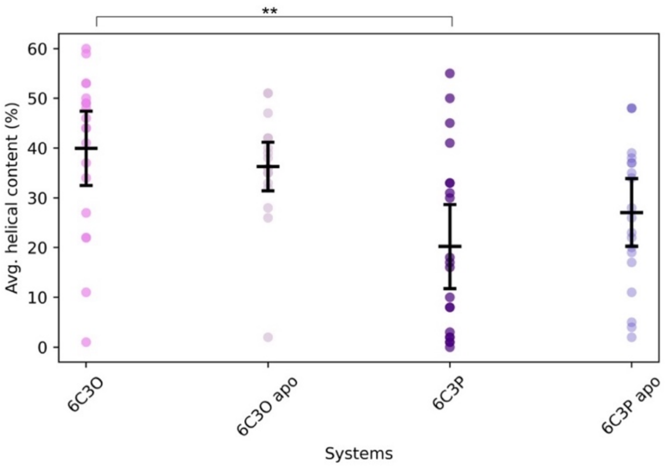

Supplementary Figure 7. Average helical content of the C-linker. The average helical content of the C-linker (residues 173-180) was calculated with DSSP (Kabsch & Sander, 1983; Touw et al., 2015) for the first 200 ns of five replicas and 4 protein subunits, resulting in 20 data points per simulation system. Helical content was calculated as a combination of 3_{10}-helices, α-helices, and π-helices. The two-sided T-test was performed with SciPy (Virtanen et al., 2020): ** $p < 0.01$

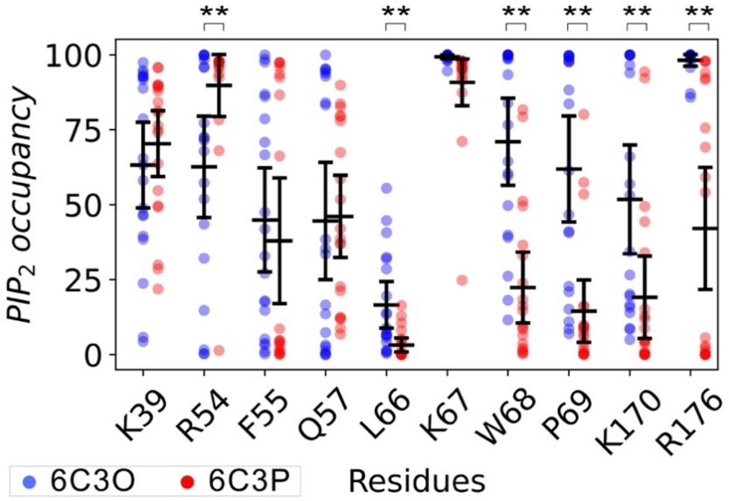

Supplementary Figure 8. Kir6.2-PIP$_2$ contact analysis. Fraction of time that Kir6.2 residues were in close proximity to PIP$_2$ headgroup atoms (cutoff 4 Å). Occupancies were calculated by parsing every 100th frame of the trajectories with PyLipid (Song et al., 2021) for all four protein subunits of 5 x 1 µs simulations, resulting in 20 data points per residue and system. The error bars indicate the 95% confidence interval around the mean. The two-sided T-test was performed with SciPy (Virtanen et al., 2020): ** $p < 0.01$

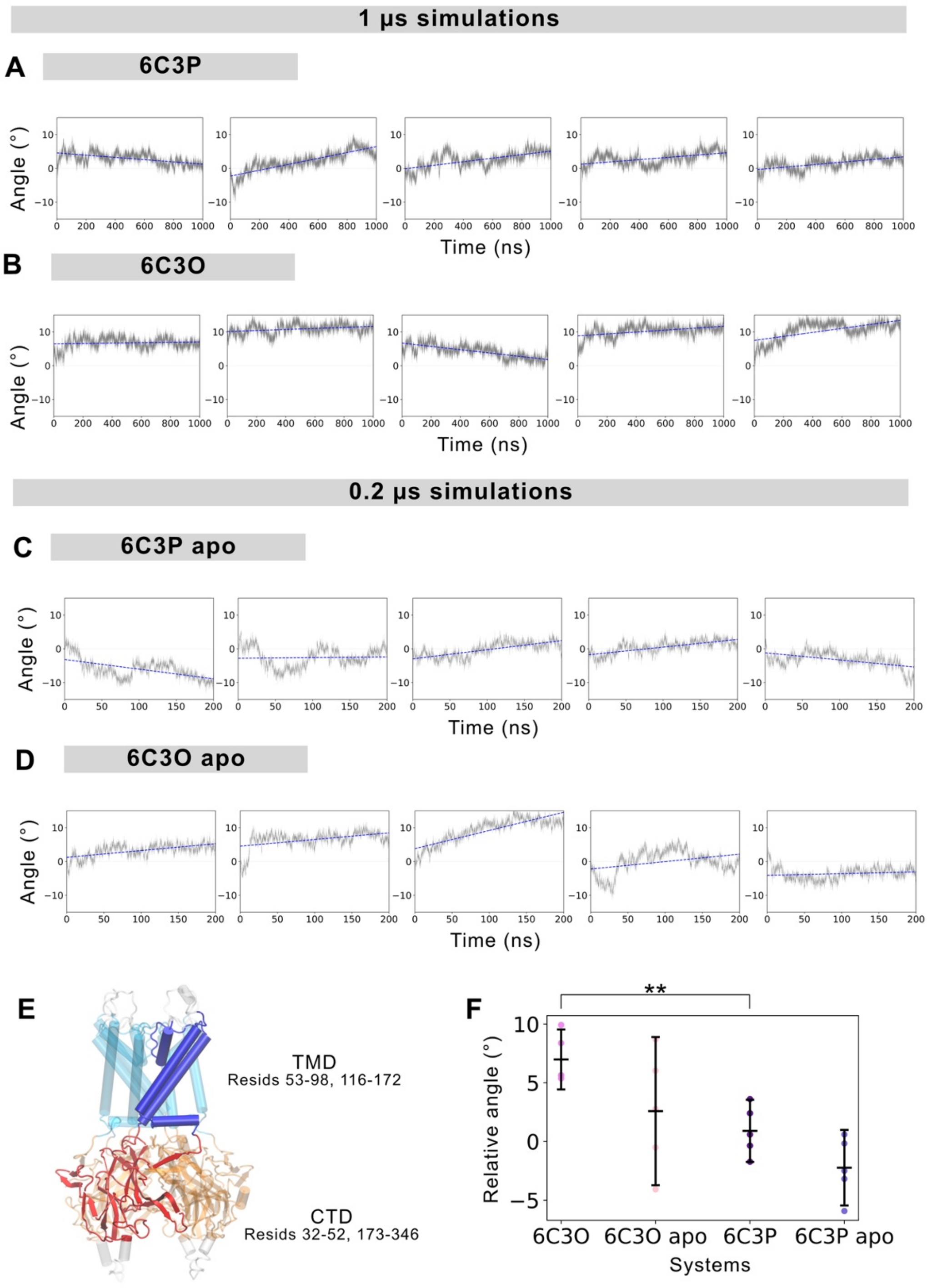

Supplementary Figure 9. Relative TMD-CTD rotation. Relative rotation angles as a function of time are shown for (A,B) 1 µs and (C,D) 0.2 µs simulations of different systems as indicated. The absolute angles were subtracted from the initial angles (6C3O: 60.76°, 6C3P: 68.24°), measured before the simulations.
(E) Schematic figure illustrating the parts of the protein that were used to calculate the center of mass (COM) for the TMD-CTD rotation, as described in *Materials and methods*. (F) For comparison, the mean values of the first 200 ns of five replicas per simulation system were calculated, resulting in 5 data points per system. The error bar indicates the 95% confidence interval around the mean. The two-sided T-test was performed with SciPy (Virtanen et al., 2020): ** $p < 0.01$

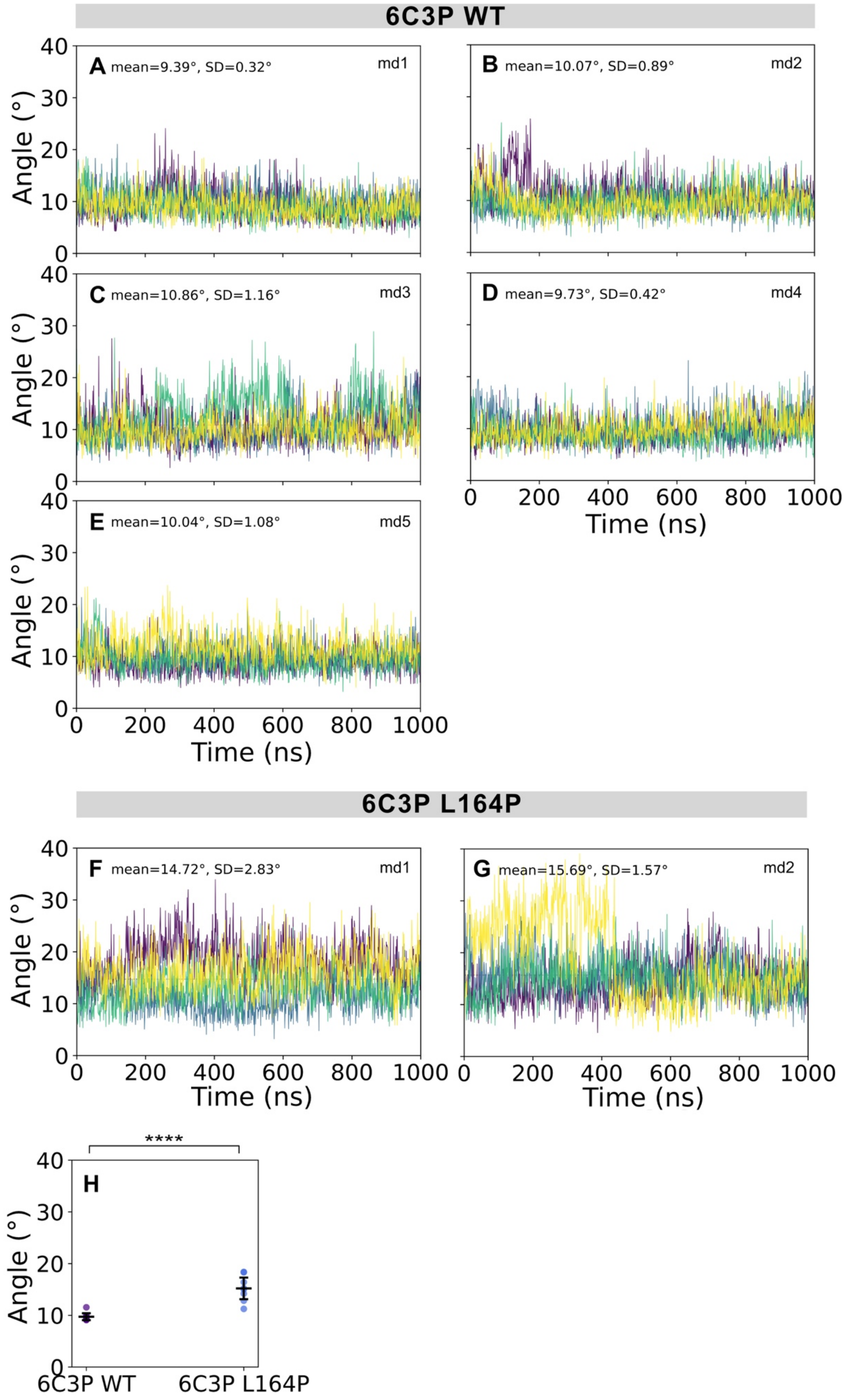
6C3P WT
A mean=9.39°, SD=0.32° md1
B mean=10.07°, SD=0.89° md2
C mean=10.86°, SD=1.16° md3
D mean=9.73°, SD=0.42° md4
E mean=10.04°, SD=1.08° md5
Angle (°)
Time (ns)
6C3P L164P
F mean=14.72°, SD=2.83° md1
G mean=15.69°, SD=1.57° md2
H
****
6C3P WT
6C3P L164P

Supplementary Figure 10. L164P induces a kink in helix M2. Maximum angles of the M2 helix (residues 143-172) were calculated with the Bendix plugin of VMD (Dahl et al., 2012) for (A-E) five 6C3P simulation replicas and (F,G) two 6C3P L164P replicas over time. The colors represent different subunits of the protein. (H) Comparison of the mean values calculated from four protein subunits of two randomly chosen 6C3P WT simulation systems (md1, md2) and both L164P mutant simulations above, resulting in 8 data points per system. The error bar indicates the 95% confidence interval around the mean. The two-sided T-test was performed with SciPy (Virtanen et al., 2020): **** $p < 0.0001$

2 Supplementary Movies

All supplementary movies show two opposing subunits of the protein tetramers in white cartoon representation. Residues forming constriction sites 1-3 are shown in gray sticks and are highlighted during the movies. Four PIP_2 molecules are shown as turquoise sticks, while water molecules are shown in surface representation. K^+ ions are represented as purple spheres.

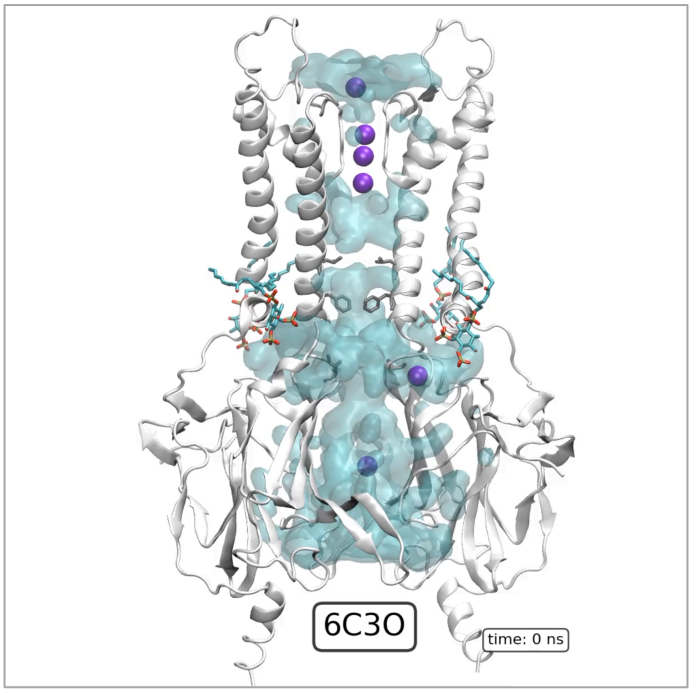

Supplementary Movie: 1. Gating and pore solvation in 6C3O

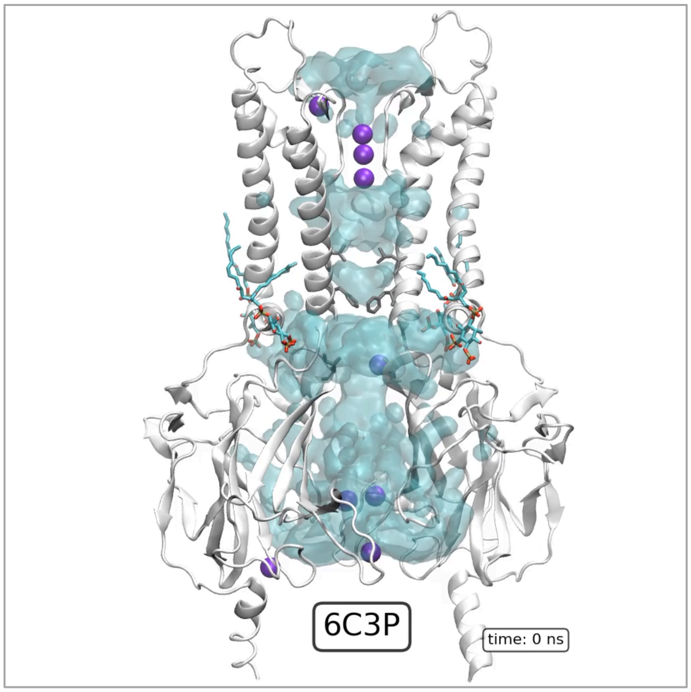

Supplementary Movie: 2. Gating and pore solvation in 6C3P

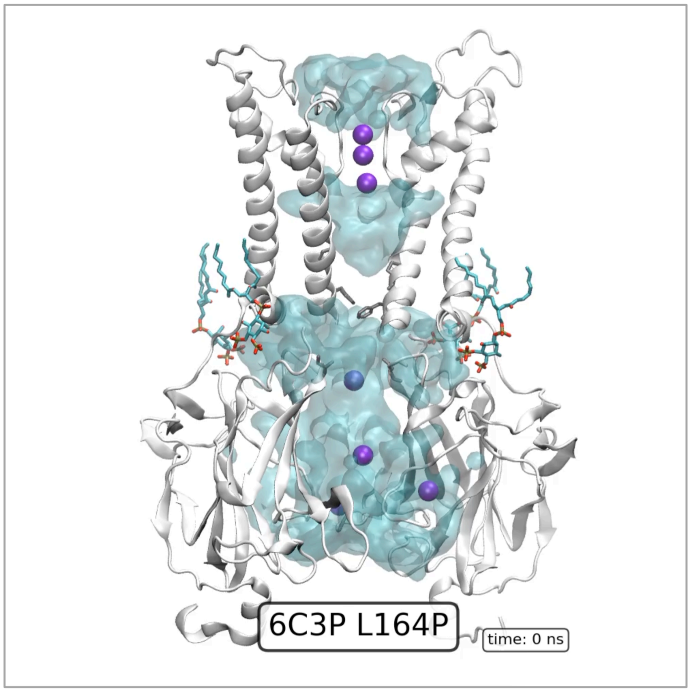

Supplementary Movie: 3. Gating and pore solvation in 6C3P L164P

3 References

Abraham, M. J., Murtola, T., Schulz, R., Páll, S., Smith, J. C., Hess, B., & Lindahl, E. (2015). Gromacs: High performance molecular simulations through multi-level parallelism from laptops to supercomputers. *SoftwareX*, *1–2*, 19–25. https://doi.org/10.1016/j.softx.2015.06.001

Abraham, M. J., van der Spoel, D., Lindahl, E., & Hess, B. (2018). *GROMACS User Manual version 2018*.

Conway, B. E. (1981). *Ionic hydration in chemistry and biophysics*. Elsevier Scientific Publishing Company, Amsterdam, Netherlands.

Dahl, A. C. E., Chavent, M., & Sansom, M. S. P. (2012). Bendix: Intuitive helix geometry analysis and abstraction. *Bioinformatics*, *28*(16), 2193–2194. https://doi.org/10.1093/bioinformatics/bts357

Guex, N., & Peitsch, M. C. (1997). SWISS-MODEL and the Swiss-PdbViewer: An environment for comparative protein modeling. *Electrophoresis*, *18*(15), 2714–2723. https://doi.org/10.1002/elps.1150181505

Hunter, J. D. (2007). Matplotlib: A 2D Graphics Environment. *Computing in Science and Engineering*, *9*(3), 90–95. https://doi.org/10.1109/MCSE.2007.55

Kabsch, W., & Sander, C. (1983). Dictionary of Protein Secondary Structure: Pattern Recognition of Hydrogen-Bonded and Geometrical Features. *Biopolymers*, *22*, 2577–2637. https://doi.org/10.1002/bip.360221211

Schrödinger, L. (2015). *The {PyMOL} Molecular Graphics System, Version~1.8*.

Song, W., Corey, R. A., Duncan, A. L., Ansell, T. B., Stansfeld, P. J., & Sansom, M. S. (2021). Pylipid: A Python Toolkit for Analysis of Lipid-Protein Interactions from MD Simulations. *Biophysical Journal*, *120*(3), 48a. https://github.com/wlsong/PyLipID

Touw, W. G., Baakman, C., Black, J., Te Beek, T. A. H., Krieger, E., Joosten, R. P., & Vriend, G. (2015). A series of PDB-related databanks for everyday needs. *Nucleic Acids Research*, *43*(D1), D364–D368. https://doi.org/10.1093/nar/gku1028

Virtanen, P., Gommers, R., Oliphant, T. E., Haberland, M., Reddy, T., Cournapeau, D., Burovski, E., Peterson, P., Weckesser, W., Bright, J., van der Walt, S. J., Brett, M., Wilson, J., Millman, K. J., Mayorov, N., Nelson, A. R. J., Jones, E., Kern, R., Larson, E., … Vázquez-Baeza, Y. (2020). SciPy 1.0: fundamental algorithms for scientific computing in Python. *Nature Methods*, *17*(3), 261–272. https://doi.org/10.1038/s41592-019-0686-2
